# The impact of panel composition and topic on stakeholder perspectives: Generating hypotheses from online maternal and child health modified‐Delphi panels

**DOI:** 10.1111/hex.13420

**Published:** 2022-01-06

**Authors:** Dmitry Khodyakov, Sujeong Park, Jennifer A. Hutcheon, Sara M. Parisi, Lisa M. Bodnar

**Affiliations:** ^1^ RAND Health Care, RAND Corporation Pardee RAND Graduate School Santa Monica California USA; ^2^ School of Public Affairs The Pennsylvania State University ‐ Harrisburg Middletown Pennsylvania USA; ^3^ Department of Obstetrics and Gynaecology University of British Columbia Vancouver British Columbia Canada; ^4^ Department of Epidemiology University of Pittsburgh Graduate School of Public Health Pittsburgh Pennsylvania USA

**Keywords:** expert panel, modified‐Delphi, online engagement, stakeholder engagement

## Abstract

**Introduction:**

Multistakeholder engagement is crucial for conducting health services research. Delphi‐based methodologies combining iterative rounds of questions with feedback on and discussion of group results are a well‐documented approach to multistakeholder engagement. This study develops hypotheses about the impact of panel composition and topic on the propensity and meaningfulness of response changes in multistakeholder modified‐Delphi panels.

**Methods:**

We conducted three online modified‐Delphi (OMD) multistakeholder panels using the same protocol. We assigned 60 maternal and child health professionals to a homogeneous (professionals only) panel, 60 pregnant or postpartum women (patients) to a homogeneous panel, and 30 professionals and 30 patients to a mixed panel. In Round 1, participants rated the seriousness of 11 maternal and child health outcomes using a 0–100 scale and explained their ratings. In Round 2, participants saw their own and their panel's Round 1 results and discussed them using asynchronous, anonymous discussion boards moderated by the study investigators. In Round 3, participants revised their original ratings. Our outcome measures included binary indicators of response changes to ratings of the low, medium and high severity maternal and child health outcomes and their meaningfulness, measured by a change of 10 or more points.

**Results:**

Participants changed 818 of 1491 (55%) of responses; the majority of response changes were meaningful. Patterns of response changes were different for patients and professionals and for different levels of outcome seriousness. Using study results and the literature, we developed three hypotheses. First, OMD participants, regardless of their stakeholder group, are more likely to change their responses on preference‐sensitive topics where there is a range of viable alternatives or perspectives. Second, patients are more likely to change their responses and to do so meaningfully in mixed panels, whereas professionals are more likely to do so in homogeneous panels. Third, the association between panel composition and response change varies according to the topic (e.g., the level of outcome seriousness).

**Conclusions:**

Results of our work not only helped generate empirically derived hypotheses to be tested in future research but also offer practical recommendations for designing multistakeholder OMD panels.

**Patient or Public Contribution:**

Pregnant or postpartum women were involved in this study.

## 1 INTRODUCTION

Multistakeholder engagement is crucial for conducting health services research; it helps ensure that key stakeholder perspectives inform the research process and its outcomes.^1^ Patients, caregivers, clinicians, researchers, payers, purchasers and policy‐makers are key stakeholders^2^ whose engagement can positively impact all stages of the research process.^3^, ^4^ Nonetheless, multistakeholder engagement is challenging due to logistical difficulties, power imbalances and stakeholders' capacity to participate meaningfully.^5^

One way to conduct multistakeholder engagement is to convene a Delphi panel.^6^, ^7^, ^8^ Delphi‐based methodologies that combine iterative rounds of questions with feedback on intermediary panel results were designed to more objectively develop group consensus.^9^, ^10^ The Delphi method is based on the idea that exposure to alternative perspectives improves the quality of the final responses, which are used to determine the existence of consensus. Delphi‐based methodologies provide a useful approach for measuring whether and how participants' perspectives change.^11^, ^12^

Modified‐Delphi methodologies that start with a survey, proceed with feedback on and an in‐person, telephone or virtual discussion of initial survey results, and end with participants revising their original survey responses offer stakeholders an opportunity to directly engage with each other, which is absent in traditional Delphi panels.^8^, ^13^, ^14^, ^15^, ^16^ Online modified‐Delphi (OMD) approaches are particularly useful engagement techniques because they allow for large‐scale (50+ participants) anonymous engagement, which is not possible in modified‐Delphi panels that meet in‐person. The requirement of in‐person discussion limits the panel size to 9–20 participants.^13^, ^14^ While the online method has clear benefits, little is known about the contextual factors such as panel composition or topic that might affect the outcomes of multistakeholder engagement.

Research suggests that stakeholder perspectives vary, with patients and clinicians, for example, having different perceptions of research priorities, treatment preferences and harm‐benefit tradeoffs.^17^, ^18^ Although patients' voices may be dominated by clinicians',^17^ true consensus in multistakeholder initiatives may not be achieved without directly exposing stakeholders to the perspectives of other groups. While patients may be more comfortable sharing their perspectives with peers and, therefore, could be more satisfied with engagement in homogeneous panels, participants in mixed panels could change their positions after being exposed to the alternative perspectives, which is key for developing true consensus in multistakeholder panels.^19^ Although it is possible to imagine how the outcomes of a multistakeholder engagement might vary depending on its topic, we are not aware of previous studies that directly addressed this question in the context of modified‐Delphi panels.

This paper advances methods for conducting online multistakeholder panels using a modified‐Delphi approach by exploring the impact of panel composition and topic on stakeholder judgments and uses the results of this analysis to generate empirically grounded hypotheses for future research. We use the propensity and meaningfulness of response changes after stakeholders receive statistical feedback and discuss their original responses with others as a measure of panel impact on individual stakeholder judgments. To reach the study goals, we use the data from three OMD panels that engaged patients and professionals around the severity of maternal and child health outcomes linked to gestational weight gain.^20^, ^21^ We treat outcomes of different levels of severity as proxies for different panel topics. Our findings have practical and methodological implications for assembling multistakeholder panels and contribute to ongoing scholarly debates about the impact of feedback^22^ and the nature of consensus‐building in Delphi panels.^11^, ^12^, ^23^

## 2 METHODS

### 2.1 Study design

In October–November 2019, we conducted three concurrent OMD panels: a panel of 60 professionals, a panel of 60 patients and a mixed panel of 30 professionals and 30 patients. This panel size is consistent with the recommendations for the optimal number of participants in each OMD panel.^24^ The study team members used their professional networks and social media, including Twitter and Facebook, to recruit 90 maternal and child health professionals who have worked in the field for at least five years and 90 patients—women who were either pregnant or gave birth in the past 2 years. Interested individuals residing in the United States were asked to complete a study registration form. We used stratified randomisation to assign participants to either homogeneous or mixed panels and ensured the desired composition of each panel.

All panels were conducted using ExpertLens™—a previously evaluated OMD platform.^13^, ^19^, ^24^, ^25^ Between October 9 and November 26, 2019, each panel completed the same three‐round OMD process. Participants were informed about the number of rounds at the recruitment stage. In Round 1, participants rated the seriousness of 11 pregnancy weight gain outcomes by entering any number between 0 (*not serious at all*) to 100 (*very serious*) and explained their responses (see Supporting Information Appendix). In Round 2, participants saw the distribution of Round 1 ratings and associated explanations, reviewed how their own ratings compared to their panel's medians and quartiles, and engaged in an asynchronous, anonymous and moderated online discussion. To preserve confidentiality, participants were only identified as ‘professionals’ or ‘patients’. The discussion lasted 2 weeks and was moderated by the same team of three study investigators in all panels. Moderators made sure that the discussion topics mentioned by panellists in one panel were not introduced by moderators in another panel, unless participants themselves raised the same issues. In Round 3, participants were allowed to revise their original ratings and were asked to assess their participation experiences with the OMD process. Participants completing all rounds received a $165 gift card.

Additional details about study design^20^ and its findings^21^ were published elsewhere.

### 2.2 Sample

Our analysis focuses on response changes to the same question between Rounds 1 and 3. We only include a participant's response to a question if it was provided in both rating rounds. Our final sample includes 143 participants and 1491 response changes.

### 2.3 Variables

The main outcome variables in this study include binary indicators of response change and its meaningfulness (Yes/No). We considered a change of 10 or more points to be meaningful because it moves a response from one decile to another on the 100‐point scale.

Our main predictor variable is the composition of the panel a participant was randomized into (patients in a homogeneous panel [reference group], professionals in a homogeneous panel, patients in a mixed panel and professionals in a mixed panel).

Our control variables include three measures of stakeholders' participation experiences, such as overall satisfaction and perceptions of the impact of two key features of the OMD panels most relevant to the goals of this study—statistical feedback as presented in charts and perceived ability of online discussions to change participant responses. Participants used 7‐point Likert scales, where 1 = *strongly disagree*, 2 = *disagree*, 3 = *slightly disagree*, 4 = *neutral*, 5 = *slightly agree*, 6 = *agree*, 7 = *strongly agree*, to rate their agreement with the following statements:

1. Participation in this study was satisfying.
2. The charts helped me understand how my responses compared to those of other participants.
3. Round 2 discussion changed my perspective on the study topics.

As in previous studies, we dichotomized responses and considered those scoring an item as 5, 6 or 7 as having positive participation experiences.^19^, ^24^, ^25^

Other control variables include participants' race (White vs. other) and age.

### 2.4 Statistical analysis

We used mixed‐effect logistic regression to estimate the panel composition effects on the presence and meaningfulness of response changes. All models were clustered at the individual level to address within‐participant correlations, and robust standard errors were produced. We first ran all the models using the seriousness ratings of all pregnancy outcomes combined (*n* = 1491 response changes). We then stratified all analyses by health outcome severity levels, which we considered as different panel topics. High severity outcomes included infant death, stillbirth, preterm birth and pre‐eclampsia (*n* = 537 response changes). Medium severity outcomes included obesity in women, childhood obesity, gestational diabetes and metabolic syndrome in women (*n* = 542 response changes). Finally, low severity outcomes included small‐for‐gestational‐age (SGA) birth, large‐for‐gestational‐age birth and unplanned caesarean delivery (*n* = 412 response changes). Additional details on outcome severity can be found elsewhere.^21^ We conducted all the analyses using STATA SE 14.

## 3 RESULTS

### 3.1 Participant characteristics

Of 180 invited participants, 143 (79%) answered at least one question in both rating rounds. Of these 143 participants, 73 (51%) were health care professionals and 70 (49%) were patients. Of 73 professionals, 46 (63%) were in the homogeneous panel and 27 (37%) were in the mixed panel. Of 70 patients, 47 (67%) were in the homogeneous panel and 23 (33%) were in the mixed panel. More detailed information on participation rates could be found elsewhere.^21^

Of 143 study participants, almost all (*n* = 131, 92%) were female and roughly two‐thirds (*n* = 93, 65%) were White (Table 1). Among 73 professionals, the majority were researchers (*n* = 56, 77%) and had a doctoral degree (*n* = 65, 93%). Two‐fifths (*n* = 29) of all professionals had 15 or more years of experience, 36% (*n* = 26) had 10–14 years, and 25% (*n* = 18) had 5–9 years of experience. The majority of 70 patients in our study had a master's or doctoral degree (*n* = 39, 61%) and reported being pregnant within the past 2 years (*n* = 53, 76%); and slightly less than half (*n* = 33, 47%) reported having two or more prior pregnancies.

**Table 1:** Participant characteristics

Participant characteristics	All respondents (*N* = 143)	All professionals (*N* = 73)	Professionals in a homogeneous panel (*N* = 46)	Professionals in a mixed panel (*N* = 27)	All patients (*N* = 70)	Patients in a homogeneous panel (*N* = 47)	Patients in a mixed panel (*N* = 23)
Demographic characteristics	*n* (%)						
Sex							
Male	12 (18)	12 (16)	8 (17)	4 (15)	0 (0)	0 (0)	0 (0)
Female	131 (92)	61 (84)	38 (83)	23 (85)	70 (100)	47 (100)	23 (100)
Hispanic ethnicity							
Yes	23 (16)	8 (11)	4 (9)	4 (15)	15 (21)	9 (19)	6 (26)
Race							
White	93 (65)	50 (68)	30 (65)	20 (74)	43 (61)	31 (66)	12 (52)
Non‐White	50 (35)	23 (32)	16 (35)	7 (26)	27 (39)	16 (34)	11 (48)
Highest education level^a^							
High school or less	7 (5)	0 (0)	0 (0)	0 (0)	7 (11)	3 (7)	4 (18)
Bachelor's degree	18 (13)	0 (0)	0 (0)	0 (0)	18 (28)	13 (31)	5 (23)
Master's degree	28 (21)	5 (7)	3 (7)	2 (8)	23 (36)	15 (36)	8 (36)
Doctoral degree	81 (60)	65 (93)	41 (93)	24 (92)	16 (25)	11 (26)	5 (23)
Age mean (SD)	39.0 (10.3)	44.7 (11.1)	44.9 (11.1)	44.3 (11.3)	33.0 (4.4)	32.8 (4.2)	33.5 (4.8)
Professional characteristics	*n* (%)						
Primary appointment	N/A				N/A		
Administrator/policy maker		1 (1)	0 (0)	1 (4)			
Health care provider		14 (19)	8 (17)	6 (22)			
Public health worker		2 (3)	1 (2)	1 (4)			
Researcher		56 (77)	37 (80)	19 (70)			
Experience							
5–9 years		18 (25)	11 (24)	7 (26)			
10–14 years		26 (36)	16 (35)	10 (37)			
15 or more years		29 (40)	19 (41)	10 (37)			
Patient characteristics	*n* (%)						
Currently pregnant	N/A				17 (24)	12 (26)	5 (22)
Previous pregnancy (20 weeks or longer) within the past 2 years					53 (76)	35 (74)	18 (78)
Number of prior pregnancies (20 weeks or more)^a^							
0					11 (16)	6 (13)	5 (22)
1					23 (33)	18 (38)	5 (22)
2 +					33 (47)	20 (42)	13 (57)
Subjective participation experiences^a^	Mean (SD)						
Participation in this study was satisfying	5.65 (1.12)	5.44 (1.08)	5.4 (1.18)	5.5 (0.91)	5.88 (1.12)	5.82 (1.19)	6 (1)
The charts helped me understand how my responses compared to those of other participants	6.39 (1.01)	6.30 (1.16)	6.3 (1.02)	6.23 (1.39)	6.49 (0.82)	6.51 (0.84)	6.43 (0.79)
Round Two discussion changed my perspective on the study topics	5.03 (1.28)	4.92 (1.26)	4.98 (1.29)	4.81 (1.23)	5.15 (1.31)	4.95 (1.35)	5.52 (1.16)

^a^ Not all respondents provided answers to all of the questions.

### 3.2 Participation experiences

Participants were generally satisfied with their study experiences (mean = 5.7, SD = 1.1), thought that the charts showing the distribution of Round 1 responses helped them understand how their responses compared to those of other participants (mean = 6.4, SD = 1.0), and felt that the discussions changed their perspective (mean = 5.0, SD = 1.3; Table 1). There were no major differences in participation experiences across panel types. Among professionals, those in the mixed panel, on average, had slightly lower scores on the questions about charts and discussions, but slightly higher scores on the overall satisfaction. Patients had slightly higher scores on all three measures of subjective participation experiences than professionals, with patients in the mixed panel being slightly more satisfied than patients in the homogeneous panel.

### 3.3 Response changes

Almost all of our 143 participants changed at least one response (*n* = 139, 97%, data not shown). Of the 1491 questions that participants answered twice, responses to 55% (*n* = 818) of all questions changed in Round 3 (Table 2). Of the 1491 responses provided twice, 563 (38%) were changed by 10 or more points (mean value of response change = 7.14, SD = 9.98; median = 5). Although the pattern of changes was similar between professionals and patients when panel type was not considered, it varied once panel type was accounted for. A higher percentage of patients' responses in the mixed panel changed (148 of 249, 59%), compared with responses provided by patients in the homogeneous panel (244 of 485, 50%). In contrast, 58% (281 of 483) of responses provided by professionals in the homogeneous panel and 53% (145 of 274) of responses in the mixed panel changed.

**Table 2:** Response change characteristics

Response change characteristics	All outcomes (*N* = 1491)	High severity outcomes (*N* = 537)	Medium severity outcomes (*N* = 542)	Low severity outcomes (*N* = 412)
	% (*n*/*N*)			
Response changed				
All respondents	55 (818/1491)	45 (240/537)	61 (330/542)	60 (248/412)
All professionals	56 (426/757)	48 (130/273)	62 (170/275)	60 (126/209)
Professionals in a homogeneous panel	58 (281/483)	49 (86/174)	67 (118/177)	58 (77/132)
Professionals in a mixed panel	53 (145/274)	44 (44/99)	53 (52/98)	64 (49/77)
All patients	53 (392/734)	42 (110/264)	60 (160/267)	60 (122/203)
Patients in a homogeneous panel	50 (244/485)	44 (76/174)	54 (96/177)	54 (72/134)
Patients in a mixed panel	59 (148/249)	38 (34/90)	71 (64/90)	72 (50/69)
Response changed meaningfully (by 10 or more points)				
All respondents	38 (563/1491)	26 (140/537)	46 (247/542)	43 (176/412)
All professionals	39 (295/757)	28 (76/273)	47 (130/275)	43 (89/209)
Professionals in a homogeneous panel	42 (202/483)	30 (53/174)	51 (91/177)	44 (58/132)
Professionals in a mixed panel	34 (93/274)	23 (23/99)	40 (29/98)	40 (31/77)
All patients	37 (268/734)	24 (64/264)	44 (117/267)	43 (87/203)
Patients in a homogeneous panel	34 (320/485)	25 (43/174)	42 (74/177)	36 (48/134)
Patients in a mixed panel	41 (103/249)	23 (21/90)	48 (43/90)	57 (39/69)

While a higher proportion of patients' responses in the mixed panel changed meaningfully (103 of 249, 41%), compared to their responses in the homogeneous panel (320 of 485, 34%), a higher proportion of professionals' responses in the homogeneous panel changed meaningfully (202 of 483, 42%), compared to their responses in the mixed panel (93 of 274, 34%). These results suggest a differential effect according to panel type. Moreover, the patterns of response changes differed by topic: A higher percentage of responses have been changed and altered by 10 or more points for medium and low severity outcomes than for high severity outcomes across all participant and panel types.

### 3.4 Model results

Table 3 shows the results of the mixed‐effects logistic regression predicting response changes. Looking at all outcomes shows that patients in the mixed panel (odds ratio [OR] = 1.5, confidence interval [CI] = 0.9–2.3) and professionals in the homogenous panel (OR = 1.4, CI = 0.9–2.1) were about 40%–50% more likely than patients in the homogeneous panel to change their ratings. These differences, however, were only marginally significant and only for patients. Moreover, panel composition was a significant predictor of response changes for medium and low severity outcomes, but not high severity outcomes. For medium severity outcomes, patients in the mixed panel (OR = 2.1, CI = 1.2–3.9) and professionals in the homogeneous panel (OR = 1.7, CI = .9–3.1) were more likely to change their ratings, compared to patients in the homogeneous panel. Moreover, patients and professionals in the mixed panel were more likely than patients in the homogeneous panel to change their answers about low severity outcomes (OR = 2.7, CI = 1.2–6.1 and OR = 1.9, CI = 0.9–3.9, respectively).

**Table 3:** Results of mixed‐effects logistic regression models predicting response changes

Predictors	All outcomes (*N* = 1461)	High severity outcomes (*N* = 528)	Medium severity outcomes (*N* = 530)	Low severity outcomes (*N* = 403)
	OR (95% CI)	OR (95% CI)	OR (95% CI)	OR (95% CI)
Patients in a mixed panel	1.5*	0.7	2.1**	2.7**
	(0.9–2.3)	(0.4–1.2)	(1.2–3.9)	(1.2–6.1)
Professionals in a mixed panel	1.1	0.9	0.9	1.9*
	(0.7–1.6)	(0.5–1.7)	(0.4–1.9)	(0.9–4.0)
Professionals in a homogeneous panel	1.4	1.0	1.7*	1.6
	(0.9–2.1)	(0.6–1.9)	(0.9–3.1)	(0.8–3.3)
Study participation was satisfying	0.8	1.2	0.5**	0.8
	(0.5–1.3)	(0.6–2.3)	(0.3–1.0)	(0.4–1.7)
The charts helped me understand how my responses compared to those of other participants	0.7	1.4	0.5	0.4*
	(0.3–1.5)	(0.6–3.7)	(0.2–1.5)	(0.1–1.2)
Round 2 discussion changed my perspective on the study topics	1.4	1.2	1.5	1.6
	(0.9–2.1)	(0.7–2.1)	(0.9–2.6)	(0.8–2.9)

*Note*: Patients in a homogeneous panel are a reference group. We control for demographic characteristics, such as race and age for all models. Models were clustered at the participant level. Coefficients for constant are excluded. Values presented in this table are odds ratios (OR) and robust 95% confidence intervals (CI).

** *p* < .05

* *p* < .1.

Those satisfied with their participation were less likely than their less satisfied counterparts to change ratings on medium severity outcomes (OR = 0.5, CI = 0.3–1.0), whereas those who felt that charts helped them understand how their responses compared to those of others were less likely to change their ratings on low severity questions (OR = 0.4, CI = 0.1–1.2). We note that small sample sizes led to imprecise estimates.

**Figure 1:**
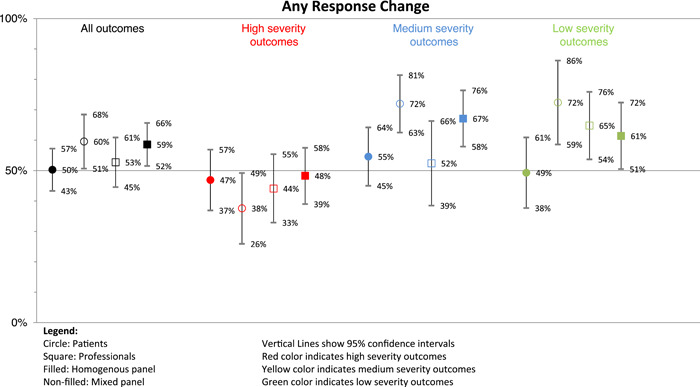
Marginal effects of the logistic regression predicting response changes

Figure 1 shows the marginal effects of the logistic regression predicting response changes, which provide additional support to our modelling results. Briefly, patients in the homogeneous panel had the lowest probability of changing their responses (50%) when looking at all outcomes together. Participants had the lowest probability (below 50%) of changing their responses on high severity outcomes. Patients in the mixed panel rating high severity outcomes had the lowest predicted probability of changing their responses (38%), whereas patients in the mixed panel rating low severity outcomes had the highest predicted probability of modifying their responses (72%).

Table 4 shows the results of the mixed‐effect logistic regression predicting the meaningfulness of response changes. Panel composition was a significant predictor of meaningful response changes only for questions about low severity outcomes. Relative to patients in the homogeneous panel, patients in the mixed panel and professionals in the homogeneous panel were more likely to meaningfully change their answers (OR = 2.5, CI = 1.2–5.3 and OR = 1.9, CI = 0.9–3.8, respectively). The difference between professionals and patients in homogeneous panels was only marginally significant.

**Table 4:** Results of mixed‐effects logistic regression models predicting meaningful response changes

Predictors	All outcomes (*N* = 1461)	High severity outcomes (*N* = 528)	Medium severity outcomes (*N* = 530)	Low severity outcomes (*N* = 403)
	**OR (95% CI)**	**OR (95% CI)**	**OR (95% CI)**	**OR (95% CI)**
Patients in a mixed panel	1.4	0.9	1.4	2.5**
	(0.8–2.3)	(0.4–1.7)	(0.7–2.8)	(1.2–5.3)
Professionals in a mixed panel	0.99	0.7	0.9	1.5
	(0.6–1.6)	(0.4–1.5)	(0.4–2.1)	(0.7–3.4)
Professionals in a homogeneous panel	1.4	1.0	1.6	1.9*
	(0.9–2.2)	(0.5–2.0)	(0.8–3.0)	(0.9–3.8)
Study participation was satisfying	0.7	0.9	0.5*	0.9
	(0.4–1.3)	(0.4–2.3)	(0.3–1.0)	(0.4–2.0)
The charts helped me understand how my responses compared to those of other participants	0.5	1.0	0.5	0.3**
	(0.2–1.2)	(0.4–2.4)	(0.2–1.6)	(0.1–1.0)
Round 2 discussion changed my perspective on the study topics	1.1	0.9	1.2	1.4
	(0.7–1.8)	(0.5–1.8)	(0.7–2.1)	(0.7–2.8)

*Note*: Patients in a homogeneous panel are a reference group. We control for demographic characteristics, such as race and age for all models. Models were clustered at the participant level. Coefficients for constant are excluded. Values presented in this table are odds ratios (OR) and robust 95% confidence intervals (CI).

** *p* < .05

* *p* < .1.

Although perceived usefulness of charts reduced the likelihood of meaningful response changes on low severity outcomes (OR = 0.3, CI = 0.1–1.0), participation satisfaction made participants marginally less likely to change their responses by 10 or more points on medium severity outcomes (OR = 0.5, CI = 0.3–1.0).

**Figure 2:**
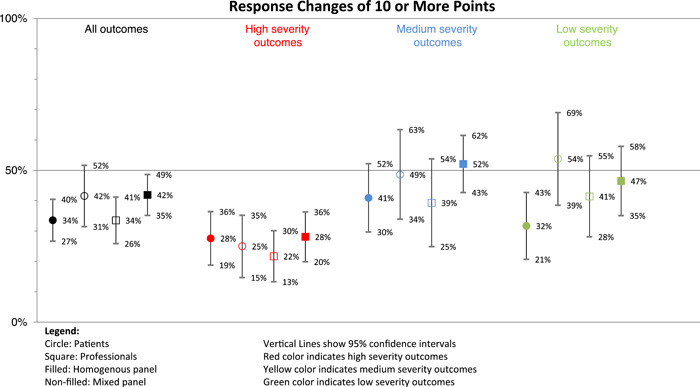
Marginal effects of the logistic regression predicting meaningful response changes

Figure 2 shows the marginal effects of the logistic regression predicting meaningful response changes. As in previous models, patients in the homogeneous panel and professionals in the mixed panel had the lowest probability of changing their responses meaningfully (34% for both groups). Looking across the outcome severity levels, the lowest predicted probability of a response change of 10 or more points was observed for high severity outcomes. Professionals in the mixed panel rating high severity outcomes was the group with the lowest probability of meaningful response changes (22%). Patients in the mixed panel rating low severity outcomes was the group with the highest probability of changing responses meaningfully (54%).

## 4 DISCUSSION

We analysed the impact of panel composition and topic on the presence and meaningfulness of response changes in OMD panels using the data on the severity of maternal and child health outcomes linked to gestational weight gain as a case study. Our results show that professionals and patients rating the seriousness of maternal and child health outcomes changed more than half of their original responses and that the majority of response changes (563 of 818, 69%) were meaningful. This finding suggests that the exposure to and the discussion of the perspective of other participants affect individual judgments about outcome severity.

In contrast to previous research that suggested that personal characteristics of OMD panellists were not associated with response changes,^12^ our results showed that participant background matters and that patterns of response changes are different for pregnant and postpartum women and maternal and child health professionals. It is worth noting, however, that the previous study focused on patient and caregiver panels on different topics, included a different set of participant background measures and did not look at the meaningfulness of change.

Our results also illustrate heterogeneity in the impacts of panel composition on response changes and their meaningfulness based on panel topic, operationalized as outcome severity. While we saw some patterns, we cannot, with certainty, say that certain types of stakeholders are more likely than others to change their responses on certain topics. Nonetheless, our study design provides a unique opportunity to generate hypotheses to be tested in future research.

First, *we hypothesize that participants in OMD panels, regardless of their stakeholder group, are more likely to change their responses for certain preference‐sensitive topics, such as those where there is a range of viable alternatives or perspectives*. In our study, the likelihood and meaningfulness of response changes were affected by the nature of maternal and child health outcomes considered. Moreover, participants had the lowest probability of changing their responses and doing so meaningfully while rating outcomes deemed ‘severe’ by participants. There is little if any debate that infant death and stillbirth are serious health outcomes. Therefore, it is not surprising that our participants rated these outcomes highly in Round 1 and that their perspectives did not change greatly after the discussion round. At the same time, individual judgments about less severe outcomes, such as gestational diabetes and SGA birth, were more affected by the perspectives of other participants. For example, while some patients may have initially focused more on the short‐term impact of gestational diabetes thinking that it resolves after delivery, exposure to a professional perspective may have brought to the fore concerns about risks of complications during delivery and increased risk of Type 2 diabetes after child‐bearing years.

Second, *we hypothesize that, regardless of the panel topic, stakeholders may be more likely to change their responses in different panel types: Patients may be more likely to change their responses and to do so meaningfully in mixed panels, whereas professionals may be more likely to do so in homogeneous panels*. The fact that patients generally had the highest probability of changing their responses in the mixed panel may be an illustration of collaborative learning, which takes place in diverse stakeholder panels that focus on important issues. Stakeholders can learn from the perspective of a different group and change their responses based on new ideas they may not have considered.^26^ Indeed, patients may be eager to learn not only from the experiences of other patients but also from professionals who have specialized expert knowledge of the topic. To illustrate, engagement with professionals could help patients learn about the potential burden of undergoing treatments for complications caused by a problem that patients may not have otherwise considered severe enough to worry about.

Although unexpected and somewhat methodologically undesirable, professionals in our study were more likely to change their responses and to do so meaningfully in the homogeneous panel. This finding, however, is not too surprising given that shared decision‐making often suffers from ‘selective paternalism’—a situation where healthcare professionals step outside of shared decision‐making to choose a course of action they think would work best for their patient, but that discounts hearing an alternative patient perspective.^27^

Third, *we hypothesize that the association between panel composition and response change may vary according to the topic*. Panel composition may play a bigger role in panels on somewhat less serious or consequential, but still important health topics. In our study, marginal effects of panel composition varied by the outcome type: While patients in the mixed panel were more likely to change their responses and to do so meaningfully on medium and low severity outcomes, the reverse was true for high severity outcomes. Professionals in the homogeneous panel generally had higher probabilities of changing their responses and doing so meaningfully than professionals in the mixed panel. This was true across topics with one exception: Professionals in the mixed panel had a higher probability of changing their responses on low severity outcomes. Although these findings support our hypothesis that patients and professionals learn under different circumstances, it offers an important nuance—mutual learning may happen in diverse panels, but only for certain types of outcomes. While previous research shows that there are no statistically significant differences between patient/caregiver and clinician/research experiences with OMD panels or their willingness to use OMD in the future,^19^ this study suggests that mixed panels may promote mutual learning in multistakeholder panels on certain topics.

Our study has important limitations. Our analysis was limited to three OMDs that engaged pregnant and postpartum women and health professionals on the topic of maternal and child health outcomes. We note that the women who participated in our study were highly educated. Therefore, the patterns of findings may be different in panels that engage different stakeholders and/or focus on other topics. Moreover, not all study participants answered the same questions twice or provided responses to satisfaction questions, which limited our sample size. Nonetheless, attrition is common in OMD panels, and our participation rates were higher than in other panels.^21^ Finally, this paper relies solely on the rating data and has not looked at how the content of online discussion comments affects response changes. Future research should test our hypotheses in OMD panels conducted with different types of participants and on different topics and analyse the impact of the discussion content on response changes in different panel types.

## 5 CONCLUSIONS

**Figure 3:**
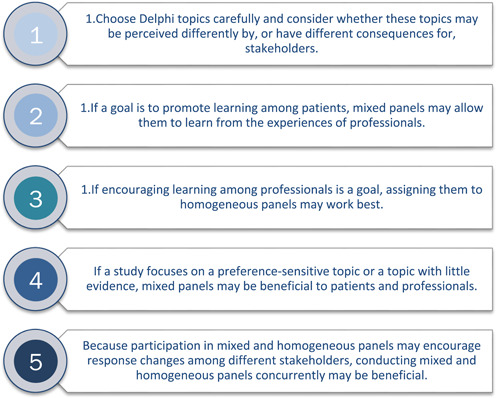
Five practical recommendations for online modified‐Delphi organizers

We recognize that our study cannot provide conclusive answers to our research questions. That is why we consider our study results, and the empirical data it relies on, as the necessary basis for formulating evidence‐informed hypotheses about panel composition and topics that should be tested in future research. Nonetheless, we believe that our results offer a number of practical recommendations, presented in Figure 3, which can help panel designers assess possible threats to achieving valid, reliable panel conclusions and encourage them to consider how panel design considerations may affect their conclusions.

## CONFLICT OF INTERESTS

Dmitry Khodyakov is a leader of the ExpertLens team. ExpertLens is a RAND‐developed online modified‐Delphi platform used to collect data in this study.

## AUTHOR CONTRIBUTIONS

All authors contributed to the study conception and design. Dmitry Khodyakov led the data collection, designed the analytic procedures and drafted the paper; Sujeong Park conducted all analyses, created all tables and figures and helped draft the paper; Jennifer A. Hutcheon and Lisa M. Bodnar obtained funding, designed the overall study, advised on the analytic procedures, reviewed and revised earlier drafts of the manuscript; Sara M. Parisi participated in the data collection and reviewed and revised earlier drafts of the manuscript. All coauthors reviewed and approved the final version of the manuscript

## Supporting information

Supporting information.

## Data Availability

Deidentified study data are available upon reasonable request from the first author.

## References

[1] Ladeji EO . Multi‐stakeholder engagement in health services research. J Comp Eff Res. 2018;7(6):517‐521. 29808715 10.2217/cer-2018-0026

[2] Deverka PA , Lavallee DC , Desai PJ , et al. Stakeholder participation in comparative effectiveness research: defining a framework for effective engagement. J Comp Eff Res. 2012;1(2):181‐194. 22707880 10.2217/cer.12.7 PMC3371639

[3] Domecq JP , Prutsky G , Elraiyah T , et al. Patient engagement in research: a systematic review. BMC Health Serv Res. 2014;14(1):89. 24568690 10.1186/1472-6963-14-89 PMC3938901

[4] Concannon TW , Meissner P , Grunbaum JA , et al. A new taxonomy for stakeholder engagement in patient‐centered outcomes research. J Gen Intern Med. 2012;27(8):985‐991. 22528615 10.1007/s11606-012-2037-1 PMC3403141

[5] Faysse N . Troubles on the way: an analysis of the challenges faced by multi‐stakeholder platforms. Nat Resour Forum. 2006;30(3):219‐229.

[6] Freeman E , Seifer SD , Stupak M , Martinez LS . Community engagement in the CTSA program: stakeholder responses from a national Delphi process. Clin Transl Sci. 2014;7(3):191‐195. 24841362 10.1111/cts.12158 PMC5350819

[7] Le Pira M , Inturri G , Ignaccolo M , Pluchino A . Modelling consensus building in Delphi practices for participated transport planning. Transp Res Proc. 2017;25:3725‐3735.

[8] Kim KK , Khodyakov D , Marie K , et al. A novel stakeholder engagement approach for patient‐centered outcomes research. Med Care. 2018;56:S41‐S47. 30074950 10.1097/MLR.0000000000000790 PMC6143220

[9] Sossa JWZ , Halal W . Zarta RHJf. Delphi method: analysis of rounds, stakeholder and statistical indicators. Foresight. 2019;21(5):525‐544.

[10] Geist MR . Using the Delphi method to engage stakeholders: a comparison of two studies. Eval Program Plann. 2010;33(2):147‐154. 19581002 10.1016/j.evalprogplan.2009.06.006

[11] Khodyakov D , Chen C . Response changes in Delphi processes: why is it important to provide high‐quality feedback to Delphi participants? J Clin Epidemiol. 2020;125:160‐161. 32413392 10.1016/j.jclinepi.2020.04.029

[12] Khodyakov D , Chen C . The nature and predictors of response changes in modified‐Delphi panels. Value Health. 2020;23(12):1630‐1638. 33248519 10.1016/j.jval.2020.08.2093

[13] Dalal SR , Khodyakov D , Srinivasan R , Straus SG , Adams J . ExpertLens: a system for eliciting opinions from a large pool of non‐collocated experts with diverse knowledge. Technol Forecast Soc Change. 2011;78(8):1426‐1444.

[14] Fitch K , Bernstein SJ , Aguilar MD , et al. The RAND/UCLA Appropriateness Method User's Manual. RAND Corporation; 2001.

[15] Grant S , Hazlewood GS , Peay HL , et al. Practical considerations for using online methods to engage patients in guideline development. Patient. 2018;11(2):155‐166. 29030831 10.1007/s40271-017-0280-6

[16] Khodyakov D , Grant S , Denger B , et al. Practical considerations in using online modified‐Delphi approaches to engage patients and other stakeholders in clinical practice guideline development. Patient. 2020;13(1):11‐21. 31544219 10.1007/s40271-019-00389-4 PMC6957573

[17] Sinha IP , Smyth RL , Williamson PR . Using the Delphi technique to determine which outcomes to measure in clinical trials: recommendations for the future based on a systematic review of existing studies. PLoS Med. 2011;8(1):e1000393. 21283604 10.1371/journal.pmed.1000393 PMC3026691

[18] Serrano‐Aguilar P , Trujillo‐Martín MM , Ramos‐Goñi JM , Mahtani‐Chugani V , Perestelo‐Pérez L , Posada‐de la Paz M . Patient involvement in health research: a contribution to a systematic review on the effectiveness of treatments for degenerative ataxias. Soc Sci Med. 2009;69(6):920‐925. 19647357 10.1016/j.socscimed.2009.07.005

[19] Khodyakov D , Grant S , Meeker D , Booth M , Pacheco‐Santivanez N , Kim KK . Comparative analysis of stakeholder experiences with an online approach to prioritizing patient‐centered research topics. J Am Med Inform Assoc. 2016;24(3):537‐543. 10.1093/jamia/ocw157 PMC7651951 28011596

[20] Bodnar LM , Khodyakov D , Himes KP , Burke JG , Parisi S , Hutcheon JA . Engaging patients and professionals to evaluate the seriousness of maternal and child health outcomes: protocol for a modified Delphi study. JMIR Res Protoc. 2020;9(6):e16478. 32222699 10.2196/16478 PMC7298634

[21] Bodnar LM , Khodyakov D , Parisi SM , Himes KP , Burke JG , Hutcheon JA . Rating the seriousness of maternal and child health outcomes linked with pregnancy weight gain. Paediatr Perinat Epidemiol. 2021;35:459‐468. 33216402 10.1111/ppe.12741 PMC8134513

[22] MacLennan S , Kirkham J , Lam TBL , Williamson PR . A randomized trial comparing three Delphi feedback strategies found no evidence of a difference in a setting with high initial agreement. J Clin Epidemiol. 2018;93:1‐8. 29017811 10.1016/j.jclinepi.2017.09.024

[23] Humphrey‐Murto S , de Wit M . The Delphi method; more research please. J Clin Epidemiol. 2019;106:136‐139. 30352274 10.1016/j.jclinepi.2018.10.011

[24] Khodyakov D , Hempel S , Rubenstein L , et al. Conducting online expert panels: a feasibility and experimental replicability study. BMC Med Res Methodol. 2011;11(1):174. 22196011 10.1186/1471-2288-11-174 PMC3313865

[25] Khodyakov D , Grant S , Barber CEH , Marshall DA , Esdaile JM , Lacaille D . Acceptability of an online modified Delphi panel approach for developing health services performance measures: results from 3 panels on arthritis research. J Eval Clin Pract. 2017;23(2):354‐360. 27619536 10.1111/jep.12623

[26] Khodyakov D , Savitsky TD , Dalal S . Collaborative learning framework for online stakeholder engagement. Health Expect. 2016;19(4):868‐882. 26295924 10.1111/hex.12383 PMC5049448

[27] Drolet BC , White CL . Selective paternalism. AMA J Ethics. 2012;14(7):582‐588. 10.1001/virtualmentor.2012.14.7.oped2-1207 23351298

