# Supplementary material for: The impact of panel composition and topic on stakeholder perspectives: Generating hypotheses from online maternal and child health modified‐Delphi panels
Source: Health Expect. 2022 Jan 6;25(2):732–43. doi: 10.1111/hex.13420 (PMC8957726; doi:10.1111/hex.13420)
Supplement: Supplementary file 1 — Supporting information. [file HEX-25--s001.docx]

**Appendix: Pregnancy Outcomes and Rating Scale Used in the Study**

| **Outcome** | **Information Provided to Participants in ExpertLens^TM^** |
| --- | --- |
| ***Stillbirth*** | **What is it?** Stillbirth is the delivery of a fetus at 20 weeks of pregnancy or later with no signs of life. It can occur before or during labor and delivery.  **What are the short-term consequences?** Parents whose baby is stillborn often experience intense grief, anxiety, fear, and suffering, and these symptoms can last for months.  **What are the long-term consequences?** Parents often remain off work for an extended period of time or reduce their working hours leading to lost wages. They may experience lingering mental health issues such as depression, anxiety, or PTSD, which may require treatment with therapy or medications. |
| ***Infant***  ***Death*** | **What is it?** Infant death is the death of a baby before his or her first birthday. Seventy percent of all infant deaths occur before 28 days of life.  **What are the short-term consequences?** Infants who survive past delivery but die later have often experienced lengthy hospital stays in the intensive care unit, potentially leading to lost wages for the parents. Parents who experience the death of their infant experience intense grief, anxiety, fear, and suffering.  **What are the long-term consequences?** Parents whose baby dies often experience trauma, suffering, grief, anxiety, sleep disturbances, and aggression. These symptoms can last for months or years. Parents often remain off work for many months or reduce their working hours. Infant death places an intense emotional strain on marriages and can lead to increased risk of divorce. Families with other children may find it difficult to care for them while experiencing grief. These other children, depending on the age, may also be emotionally affected by the infant death. |
| ***Gestational***  ***Diabetes*** | **What is it? Gestational diabetes is a pregnancy disorder that disrupts the way a woman’s body uses sugar and leads to high blood sugar levels.**  **What are the short-term consequences? Women diagnosed with gestational diabetes are required to regularly check blood sugar up to 4 times per day, modify their diet, increase their physical activity, and in some cases get insulin injections when the condition can’t be managed by diet and exercise alone. Gestational diabetes can make the baby grow too large to fit through the birth canal, which may cause the mother to have a cesarean delivery (c-section). It can also lead to jaundice, breathing problems, and low blood sugar upon birth for the baby, all of which may lead to stays in the neonatal intensive care unit. Gestational diabetes also increases the mother’s chance of having preeclampsia, a rare but serious problem of high blood pressure during pregnancy.**  **What are the long-term consequences? While gestational diabetes typically goes away once the women delivers the baby, the woman is at increased risk of developing gestational diabetes in a future pregnancy. Women with gestational diabetes are also at increased risk of developing Type 2 diabetes after their child-bearing years are over. Their babies who experienced gestational diabetes in the womb are also at increased risk of developing Type 2 diabetes later in life.** |
| ***Preeclampsia*** | **What is it? Preeclampsia is a condition in which the pregnant woman develops high blood pressure. She may also have too much protein in her urine, or problems with organs like the liver, kidney, brain, or eyes.**  **What are the short-term consequences? A pregnant woman diagnosed with preeclampsia without severe features may be admitted to the hospital for close monitoring. If she is managed at home, she will need to check her blood pressure at least daily and visit her provider’s office at least once a week. She will also need frequent fetal heart rate monitoring and ultrasounds, as well as regular blood tests. There is no cure for preeclampsia except for delivering the baby. Families with other small children at home may need to make emergency childcare arrangements when the pregnant woman is admitted to the hospital due to preeclampsia complications. Most preeclampsia cases resolve without major problems, although symptoms of preeclampsia can last for up to 6 weeks postpartum. In about 1% of preeclampsia cases, a woman may suffer serious complications or die. In some cases, babies born to mothers with preeclampsia may not grow well and be born too small. These babies are also at increased risk of being born too early (preterm birth), dying in the womb (stillbirth), or dying after delivery (infant death).**  **What are the long-term consequences? Women who develop preeclampsia are 2 to 3 times as likely as those without the disease to have heart disease, stroke, or diabetes later in life.** |
| ***Preterm***  ***Birth*** | **What is it? Preterm birth is the delivery of a baby occurring between 20 and 37 weeks of pregnancy (pregnancy normally lasts about 40 weeks).**  **What are the short-term consequences? Preterm birth is the 2nd leading cause of infant death. About 3.5% of preterm infants die before their first birthdays. Infants who survive after a preterm birth often go the neonatal intensive care unit. Short term complications include bleeding in the brain, lung failure, or infections of the intestine. The earlier the baby is born, the greater the risk of these complications and the longer the stay in the intensive care unit. Parents may experience lost wages, reduce hours or need to quit their job to manage their child’s complicated schedule of intervention therapies, specialist visits and follow-up surgeries within the first few years of their life.**  **What are the long-term consequences? Many complications can persist throughout the child’s life including problems related to their learning, speech, hearing, vison, and motor skills. Babies who had breathing problems upon birth are at increased risk of asthma later. These long-term conditions may necessitate regularly visiting many medical specialists throughout the child’s life, leading to missed school and work. Babies born preterm are more likely to develop heart disease, high blood pressure and diabetes as adults. Mothers who deliver preterm are about 50% more likely than women who deliver at term to experience heart disease and stroke up to 35 years after the birth.** |
| ***Unplanned***  ***Cesarean***  ***Section*** | **What is it? An unplanned cesarean delivery (c-section) is a surgery to deliver the baby that happens during labor (as opposed to a planned c-section which takes place when the woman isn’t in labor).**  **What are the short-term consequences? There are more complications from an unplanned c-section than from a scheduled, pre-labor c-section or from a vaginal delivery. Rarely, women with an unplanned c-section develop an infection, have excessive bleeding, receive an injury to internal organs, develop blood clots, or experience problems related to anesthesia. These conditions may lead to re-admission to the hospital after delivery. This can be difficult to manage for families with older children. Babies born from c-sections may be more likely to experience breathing problems upon delivery and are at low risk for injuries from nicks or scrapes from the procedure.**  **What are the long-term consequences? There are no established long-term consequences of an unplanned c-section.** |
| ***Small-for-gestational-age*** | **What is it? A small-for-gestational-age (SGA) birth is an infant who weighs less at birth than what is normal for their gestational age.**  **What are the short-term consequences? Most SGA infants are small but healthy, but roughly 1 in 3 SGA infants did not grow appropriately in the womb. Many SGA babies go to the neonatal intensive care unit due to low blood sugar, jaundice, feeding difficulties, and inability to regulate their body temperature. Most of these problems resolve in days to months. Mothers of SGA babies stay in the hospital about a day longer than mothers of babies who weigh within the normal range.**  **What are the long-term consequences? SGA babies are about 30% more likely than other babies to develop obesity, diabetes, and heart disease as adults.** |
| ***Large-for-gestational age*** | **What is it? A large-for-gestational-age (LGA) birth is an infant who weighs more at birth than what is normal for their gestational age.**  **What are the short-term consequences? Most of these infants are large but healthy. About 30% of LGA babies go to the neonatal intensive care unit and are at risk low blood sugar and lung problems. Most of these problems resolve in days to months. The baby may be too large to fit through the birth canal, which may cause the mother to have a cesarean delivery (c-section). Rarely LGA babies have a birth injury related to their size.**  **What are the long-term consequences? It is possible that some LGA infants have a higher risk of becoming obese or having diabetes in childhood.** |
| ***Metabolic***  ***Syndrome*** | **What is it? Metabolic syndrome is a cluster of medical problems linked to increased risk of chronic disease. To be diagnosed with metabolic syndrome, a person must have at least 3 of 5 of the following conditions: excess fat stored around the waist (i.e., “high waist circumference” or “apple-shaped”), high blood pressure, high blood sugar, high triglycerides (a fat-like substance in the blood), or low HDL cholesterol (“good” cholesterol).**  **What are the short-term consequences? Managing metabolic syndrome involves making extensive dietary changes and increasing physical activity. It may also include taking medications to reduce blood pressure, cholesterol, and blood sugar. Women with metabolic syndrome who become pregnant are at increased risk of developing gestational diabetes and preeclampsia while pregnant.**  **What are the long-term consequences? Women with metabolic syndrome are 2 times as likely as those without it to then develop heart disease, Type 2 diabetes, or stroke.** |
| ***Childhood***  ***Obesity*** | **What is it? Obesity in children is a state of excess body fat experienced during childhood. Conditions that affect the mother during pregnancy (such as maternal obesity, gestational diabetes, and high blood pressure) lead to an increased risk of childhood obesity for that baby.**  **What are the short-term consequences? Obesity in children is associated with complex medical problems including asthma, sleep problems, high blood pressure, diabetes, heartburn, and joint problems. These children are also at higher risk of anxiety and depression, as well as social problems such as bullying. Managing obesity in children requires extensive diet changes and increased physical activity. It may also require nutritional counseling, visits to specialists and therapy for the child and the caregivers, which can lead to missed school and work.**  **What are the long-term consequences? About 1 in 3 preschool-aged children with obesity and 1 in 2 school-aged children with obesity will to go on to have obesity as an adult, putting them at higher risk of diabetes, heart disease, cancer, stroke, gallbladder disease, and arthritis.** |
| ***Maternal***  ***Obesity*** | **What is it? Obesity in women is a state of excess body fat experienced during adulthood and is considered to be a chronic disease. After having a baby, women are at increased risk for developing obesity due to weight retention from the pregnancy. This risk increases with each subsequent pregnancy.**  **What are the short-term consequences? Pregnant women with obesity are 1.5 to 2 times as likely as those without obesity to experience a stillbirth or infant death, gestational diabetes, preeclampsia, cesarean delivery, or a preterm birth. They are 3 times as likely to have a child with obesity.**  **What are the long-term consequences? Obesity in adulthood often leads to high blood pressure, dyslipidemia (high levels of lipids in the blood), heart disease, stroke, diabetes, cancer, asthma, abnormal sleep, gallbladder disease, arthritis, and difficult physical functioning. People with obesity can experience discrimination because of their weight and may experience mental health problems related to this discrimination.** |

This table originally appeared in Appendix S1 in Bodnar et al (2020)

**Rating Criterion:**

Participants were asked to rate each health outcome on a scale from 0 (not serious at all) to 100 (very serious) by entering any number from 0 to 100 in a space provided:

How serious is this outcome? _____ 0 (not serious at all) to 100 (very serious)
